# An esthetic, functional, and low‐invasive lower red lip reconstruction using a mucosal perforator flap: A case report

**DOI:** 10.1002/ccr3.7499

**Published:** 2023-06-09

**Authors:** Sho Yamakawa, Shota Suda, Kenji Hayashida

**Affiliations:** ^1^ Department of Surgery Social Medical Corporation Sekisyukai Muikaichi Hospital Muikaichi Japan; ^2^ Division of Plastic and Reconstructive Surgery Faculty of Medicine Shimane University Izumo Japan

**Keywords:** island flap, lip reconstruction, low‐invasive surgery, mucosal perforator flap, venous malformation

## Abstract

**Key Clinical Message:**

Lower red lip reconstruction using a mucosal perforator flap is low‐invasive and adheres to the concept of “like with like.” The location of the mucosal perforator can easily be detected using color Doppler ultrasound.

**Abstract:**

Lip reconstructions should provide results of a high degree regarding both functionality and esthetics. We describe a case of lower red lip reconstruction using a mucosal perforator. An 81‐year‐old man complained of repeated bleeding from a submucosal venous malformation on his lower red lip, and surgery was performed under local anesthesia. The venous malformation was completely resected. A 4 cm × 2 cm triangle‐shaped flap containing a mucosal perforator, identified using color Doppler ultrasound preoperatively, was designed in the lower red lip adjacent to the defect. The perforator flap was raised in the submucosal layer, and the defect was covered with the flap in an advancement manner. The flap transfer‐related defect was closed, and at the one‐year follow‐up examination, no recurrence, drooling, or speech impediment was observed. In this case, excellent functional and esthetic results were achieved following the low‐invasive reconstruction using a mucosal perforator flap.

## Graphical abstract

A triangle‐shaped flap containing a mucosal perforator was designed in the lower red lip adjacent to the defect. The perforator flap was raised in the submucosal layer, and the defect was covered with the flap in an advancement manner.

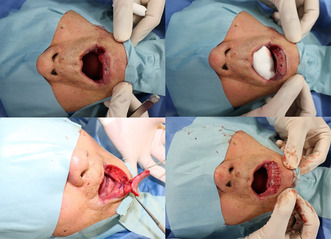

## 1 BACKGROUND

Facial reconstructions, especially for the treatment of nonmalignant diseases such as a cleft lip or a benign tumor, are focused on achieving both functional and esthetic results to a high degree.^1^ In particular, free edges such as the lips and eyelids, which involve movement, can easily become dysfunctional without high‐quality reconstruction, adversely affecting the patient's quality of life.^2^, ^3^ “Like with like” reconstruction is one of the fundamental principles of tissue reconstruction, and the best functional and esthetic results are achieved by reconstructing the defect with a similar tissue.^4^ Recently, many well‐known and unknown cutaneous perforator flaps have been used for facial reconstructions with a certain degree of success.^3^, ^5^, ^6^ However, to the best of our knowledge, there are no reports regarding red lip reconstruction using an island mucosal perforator flap. In this report, we describe a case of red lip reconstruction using a mucosal perforator arising from vessels running parallel to the lower lip vermilion.

## 2 CASE PRESENTATION

**FIGURE 1:**
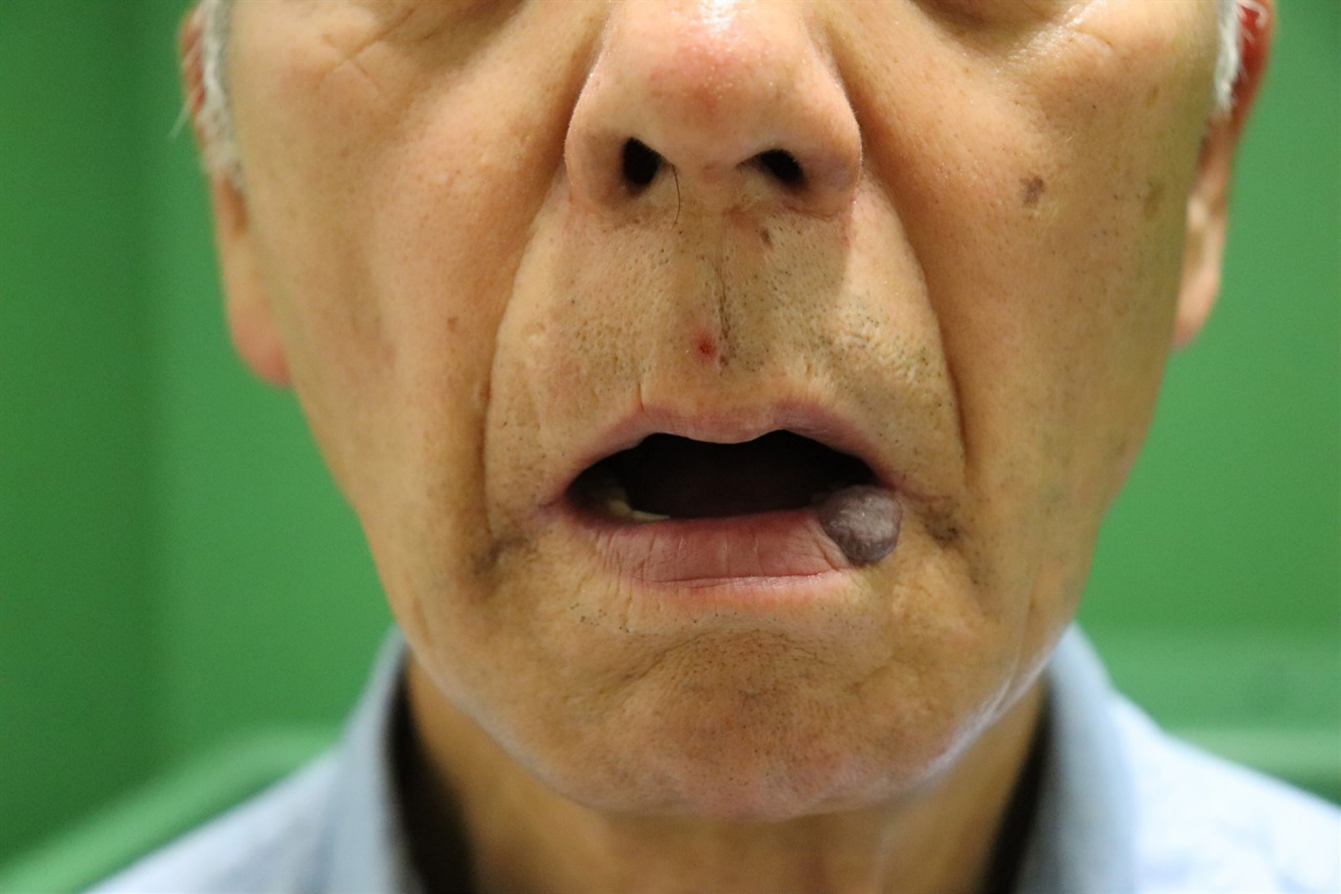
An 81‐year‐old man presented with a 2 cm × 2 cm × 1 cm venous malformation on the lower lip at the left corner of the mouth.

**FIGURE 2:**
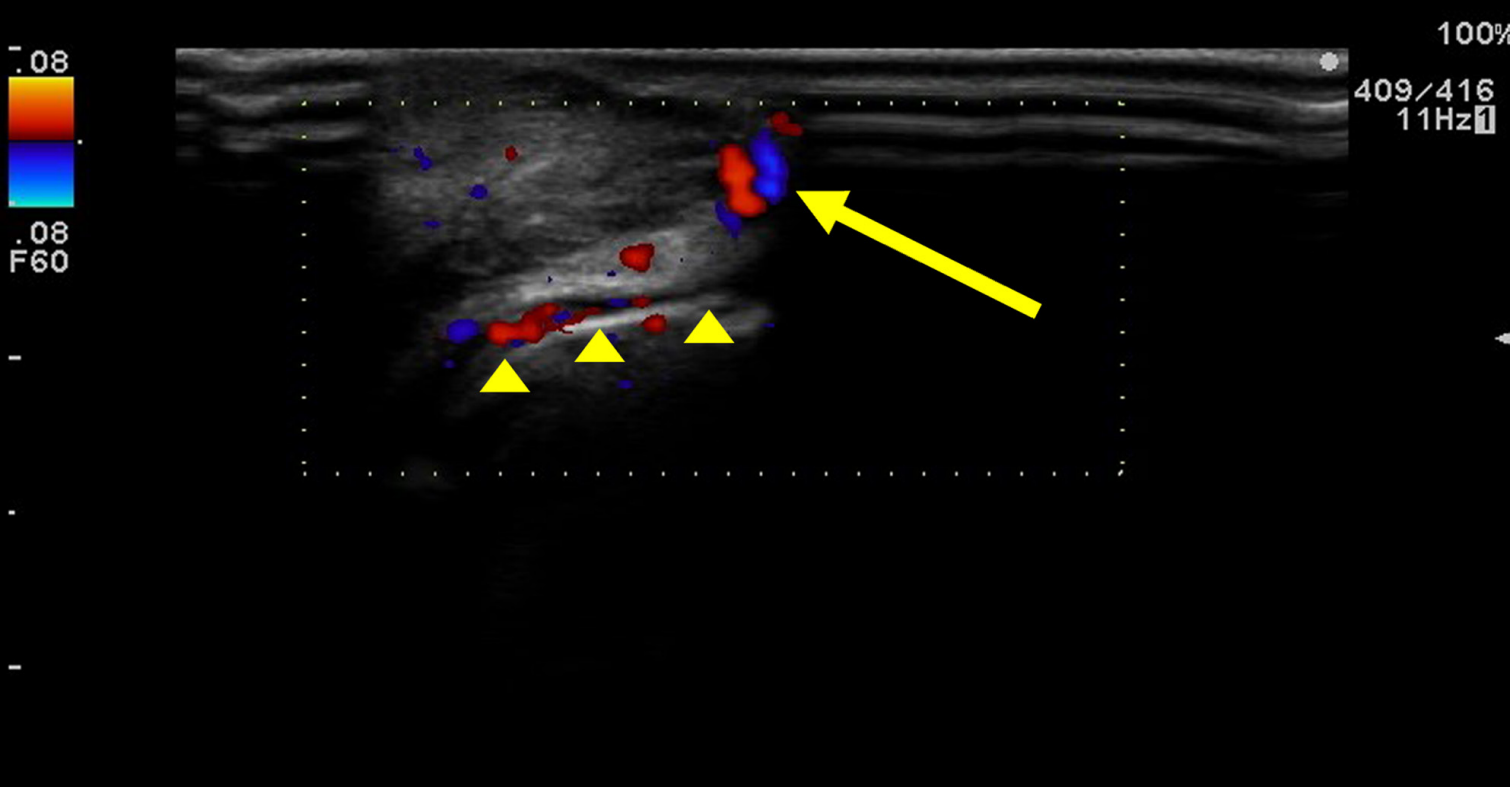
A mucosal perforator arising from a blood vessel running through the orbicularis oris muscle was identified on the center of the lower red lip and marked using a color Doppler ultrasound. Arrowheads indicate the blood vessel running through the orbicularis oris muscle. The arrow indicates the mucosal perforator rising from the blood vessel.

An 81‐year‐old man presented to our hospital with repeated bleeding from a submucosal mass on his lower lip that had appeared 2 years prior. Upon initial examination, although no ulcer was present, a 2 cm × 2 cm × 1 cm mass was observed on the patient's lower red lip at the left corner of his mouth (Figure 1). Color Doppler ultrasound (ARIETTA; Hitachi Ltd.) showed a mass filled with blood in the cavity. Therefore, a venous malformation was diagnosed, and the lesion was localized. Accordingly, resection and reconstruction with a local flap were planned. Preoperatively, on the center of the lower red lip, a mucosal perforator arising from vessels running through the orbicularis oris muscle was identified and marked using color Doppler ultrasound (Figure 2).

**FIGURE 3:**
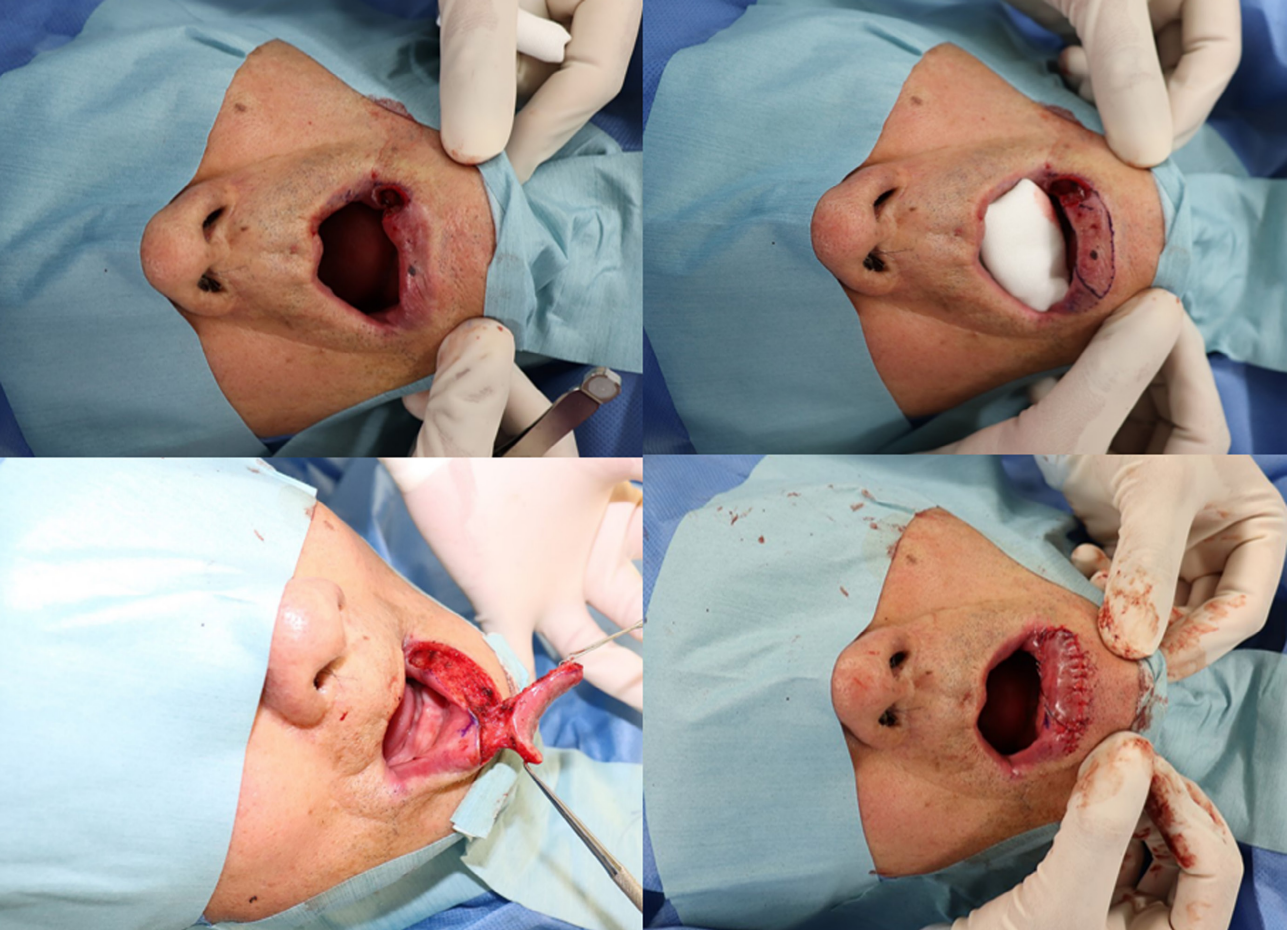
The venous malformation was resected with no margin (top left). A 4 cm × 2 cm triangle‐shaped flap containing a mucosal perforator in the lower red lip was designed adjacent to the defect (top right). The island flap was incised and dissected while preserving the mucosal perforator as a vascular pedicle (bottom left). The defect was covered by transferring the flap in an advancement manner, and the flap transfer‐related defect was closed (bottom right).

Surgery was performed under local anesthesia, and the venous malformation was completely resected with no margin (Figure 3, top left). A 4 cm × 2 cm triangle‐shaped flap containing a mucosal perforator in the lower red lip was designed adjacent to the defect (Figure 3, top right). The anterior incision line of the flap was designed to match the vermilion border of the lower lip, and the posterior incision line was designed to match the posterior edge of the defect. Subsequently, the posterior side of the flap was incised and raised in the submucosal layer, in a direction from the edge of the defect to the right, until the perforator was observed. The anterior side of the flap was then incised into an island shape and dissected while preserving the perforator as a vascular pedicle (Figure 3, bottom left). The defect was subsequently covered by transferring the flap in an advancement manner, and the flap transfer‐related defect was closed. The mucosa was sutured (Polysorb™ Size 4–0; Covidien Inc.) (Figure 3, bottom right). The postoperative course was uneventful, and the mucosa sutures were removed on postoperative day 8.

**FIGURE 4:**
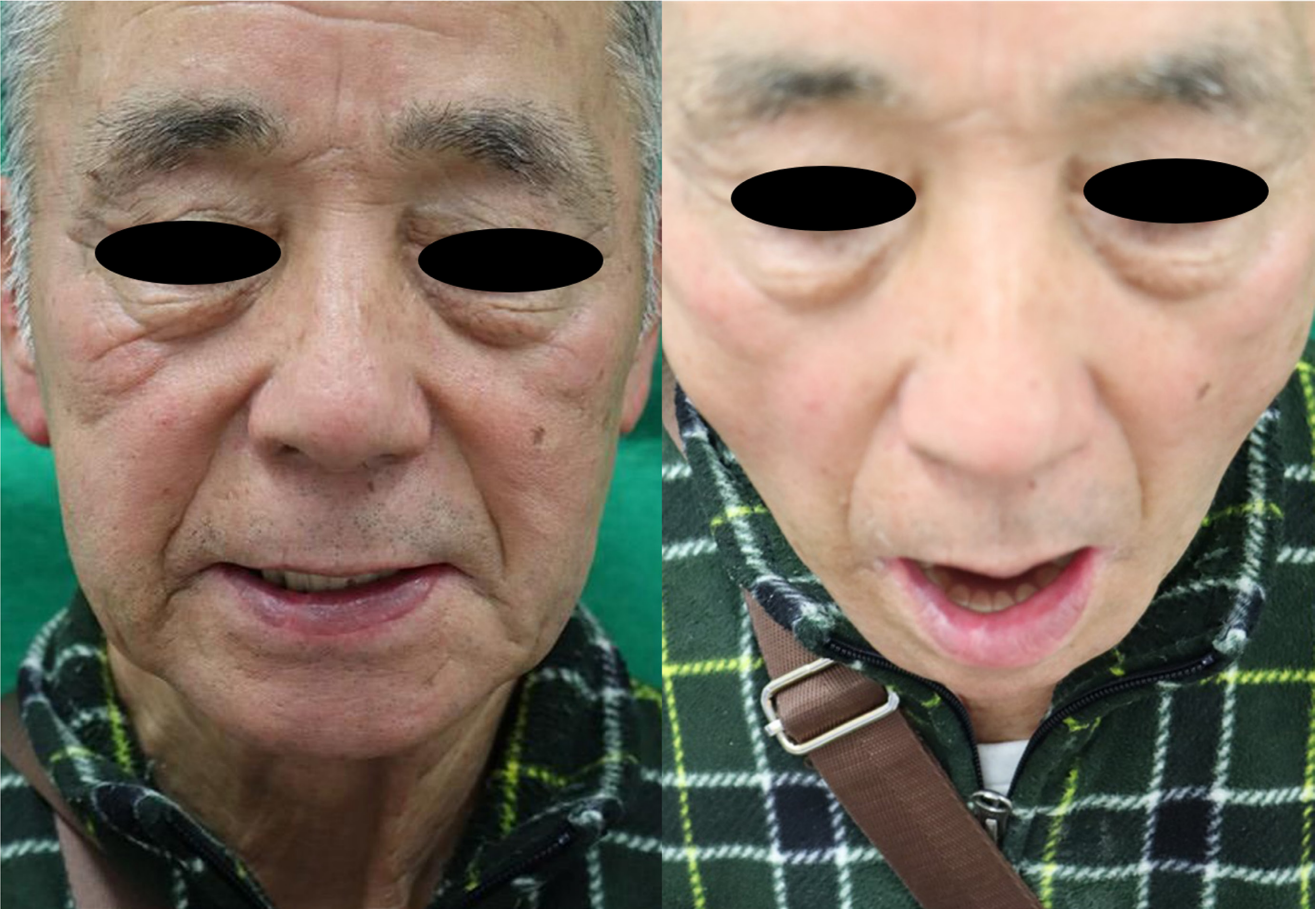
Images obtained 1 year postoperatively showed the symmetry of the reconstructed lower lip and the inconspicuous appearance of the scar.

At the 1‐year follow‐up examination, no recurrence, drooling, or speech impediment was observed. Although the patient had complained of mild hypoesthesia in the lower lip, he was satisfied with the lower lip symmetry and the inconspicuous appearance of the scar (Figure 4).

## 3 DISCUSSION

The lower lip is not only important regarding function, such as feeding and speech, but it is also a component of the face and, therefore, must be reconstructed with a good esthetic appearance.^7^ Traditionally, various techniques have been used to reconstruct the lower lip, ranging from simple direct closures to local or distant flaps, including vermilion advancement flaps and Abbe and Estlander flaps.^8^ The appropriate reconstructive technique is selected according to the defect in each case; for example, a simple direct closure is appropriate for a relatively small defect, affecting less than 1/3 of the lip, while the Abbe‐Estlander flap is useful for the reconstruction of a relatively large defect, affecting 1/3–2/3 of the lip, although there is no “gold standard.”^9^ Therefore, modified conventional reconstructive techniques have also been reported.^2^, ^10^ In 2009, Oki et al. reported a case of one‐stage reconstruction of the upper lip using an inferior labial artery pedicled island flap, a modification of the Abbe flap, which generally requires two surgeries.^10^ Although the defect in this case was small enough to consider a simple direct closure, this would have required unnecessary excision of the orbicularis oris muscle and lower white lip skin to avoid a dog‐ear deformity. In the present partial defect reconstruction, it was important to avoid functional sacrifice, and thus, a minimally invasive reconstruction using a mucosal perforator flap was ideal. We reconstructed the lower lip functionally and esthetically, owing to the preservation of vital tissues, including the orbicularis oris muscle and the white lip skin. The mucosal perforator flap, which was a vascular pedicled island flap, improved flap mobility compared with conventional flaps such as the vermilion advancement flap.^11^ Consequently, contracture deformity caused by flap retroversion was avoided.

To the best of our knowledge, no previous reports of lower lip reconstruction using a mucosal perforator flap exist. Although the term “perforator” was not used, the lower red lip reconstruction reported by Suda et al., which involved a 180‐degree rotation of the submucosal tissue as a pedicle, is presumed to be similar to the current approach using a perforator.^12^ Based on a cadaveric study, Coronel‐Banda et al. reported that the average number of mucosal perforators arising from a single facial artery was 5.2, which were 0.5 mm in diameter and 1.6 cm in length.^13^ When compared to facial artery cutaneous perforators, mucosal perforators were found to be inferior regarding number, diameter, and length; however, both mucosal and cutaneous perforators were present.^13^ Although the study did not directly evaluate the mucosal perforator of the lower lip, a study conducted by Lee et al., using 63 hemifaces of cadavers, reported that the vessels running through the deep central portion of the lower red lip included the horizontal labiomental artery or inferior labial artery, both of which were of facial artery origin.^14^ Moreover, another study reported that in 92.3% of cases, at least one mucosal perforator was present in the central red lip.^14^ Therefore, this reconstructive procedure using the mucosal perforator in the central part of the red lip can be safely performed by preoperatively confirming the location and direction of the perforator by color Doppler ultrasound.

In this study, we reported a lower red lip reconstruction case using a mucosal perforator flap. We performed an esthetic, functional, and low‐invasive lower red lip reconstruction using a mucosal perforator arising from vessels that run parallel to the vermilion border of the lower lip.

## AUTHOR CONTRIBUTIONS

**Sho Yamakawa:** Conceptualization; methodology; project administration; writing – original draft. **Shota Suda:** Resources. **Kenji Hayashida:** Supervision; writing – review and editing.

## FUNDING INFORMATION

No funding was obtained for this study.

## CONFLICT OF INTEREST STATEMENT

The authors have no competing interests to disclose.

## ETHICS STATEMENT

This case report did not receive ethical approval.

## CONSENT

The patient provided written informed consent to participate in this study and for publication of this case report and accompanying images. A copy of the written consent is available for review by the Editor of this journal.

## Data Availability

Data sharing is not applicable to this article as no datasets were generated or analyzed during the current study.
